# Lifestyle interventions after colorectal cancer surgery using a mobile digital device: A study protocol for a randomized controlled trial

**DOI:** 10.1097/MD.0000000000031264

**Published:** 2022-10-14

**Authors:** Young Il Kim, In Ja Park, Chan Wook Kim, Yong Sik Yoon, Seok-Byung Lim, Chang Sik Yu, Jin Cheon Kim, Yura Lee, Harin Kim, Seockhoon Chung, Chang-Min Choi, Hui Jeong Lee, Kyung Won Kim, Yousun Ko, Sung-Cheol Yun, Min-Woo Jo, Jong Won Lee

**Affiliations:** a Division of Colon and Rectal Surgery, Department of Surgery, Asan Medical Center, University of Ulsan College of Medicine, Seoul, Korea; b Department of Information Medicine, Asan Medical Center, University of Ulsan College of Medicine, Seoul, Korea; c Department of Psychiatry, Asan Medical Center, University of Ulsan College of Medicine, Seoul, Korea; d Department of Pulmonology and Critical Care Medicine, Asan Medical Centre, University of Ulsan College of Medicine, Seoul, Korea; e Division of Breast Surgery, Department of Surgery, Asan Medical Center, University of Ulsan College of Medicine, Seoul, Korea; f Department of Radiology and Research Institute of Radiology, Asan Medical Center, University of Ulsan College of Medicine, Seoul, Korea; g Biomedical Research Center, Asan Institute for Life Sciences, Asan Medical Center, Seoul, Korea; h Division of Epidemiology and Biostatics, Clinical Research Center, Asan Medical Center, Seoul, Korea; i Department of Preventive Medicine, University of Ulsan College of Medicine, Seoul, Korea.

**Keywords:** colon cancer, mobile apps, quality of life, rectal cancer

## Abstract

In treating colorectal cancer, surgical techniques and adjuvant treatments have advanced over the past century, but relatively less attention has been given to improve health-related quality of life (HRQOL). Recent studies report a significant association between cancer recurrence and patient lifestyle after surgery, hence emphasizing the need to assist patients to reduce this risk through appropriate lifestyle choices. The proposed study will evaluate the effects of digital interventions on lifestyle after surgery for colorectal cancer using mobile applications.

**Methods:**

A randomized controlled trial design was proposed. A total of 320 patients diagnosed with colorectal cancer aged between 20 and 70 years were to be enrolled and randomized in equal numbers into 4 groups (3 groups assigned to different mobile applications and a control group). Surveys that evaluate HRQOL, physical measurements, and metabolic parameters (fasting glucose, hemoglobin A1C, triglyceride, high-density lipoprotein cholesterol), and fat/muscle mass measurements by abdominal computed tomography (CT), will be conducted prior to surgery and every 6 months post-surgery for 18 months. Statistical analysis will be used to compare the outcomes between groups.

**Discussion:**

Results from this study could provide evidence that easily accessible mobile applications can influence patient lifestyles. Results showing minimal effects of such applications could also be constructive for improving healthcare-related applications.

## 1. Introduction

Colorectal cancer is the third most common cancer worldwide, with 1.9 million new cases reported in 2020.^[1]^ In treating these tumors, surgical techniques and adjuvant therapies have advanced much in recent decades. Combination chemotherapies and/or radiotherapy with groundbreaking surgical concepts such as total mesorectal excision or complete mesocolic excision have resulted in remarkable improvements in survival outcomes for these patients.^[2–6]^ Currently also, surgeons mainly perform oncologically feasible resections using minimally invasive surgeries with laparoscopic or robotic systems.^[7]^

The desire to improve patient survival outcomes has led to these treatment advances. However, relatively less attention has been given to the quality of life of colorectal patients after surgery. After bowel resection and anastomosis, patients can experience lifestyle changes related to bowel habits and diet. This can lead to unmet needs in colorectal cancer survivors and a poor quality of life.^[8]^ In addition, the importance of dietary control and exercise to increase muscle mass has been emphasized in recent compelling studies. Physical activity has been reported to reduce recurrence, whereas a high-insulinogenic diet was found to increase the risk of recurrence and mortality, in colon cancer survivors.^[9,10]^ Sarcopenia has also emerged as an important prognostic factor for various cancers and is reported to be associated with a poorer survival outcome in both colon and rectal cancer.^[11,12]^

We speculated that regulating a patient’s lifestyle after colorectal cancer surgery may help to further improve survival outcomes and also the health-related quality of life (HRQOL). However, this would be difficult to achieve with the routine post operative management protocols used currently in these cases. Follow-ups are recommended every 3 to 12 months depending on the cancer stage, specific situation for individual patients, and in accordance with different guidelines.^[13–15]^ Evaluating the everyday lifestyle of these patients and providing individualized advice would likely be very challenging. To overcome the time and distance obstacles to conducting such lifestyle assessments, many clinicians are attempting to utilize portable digital technologies such as a cellular phone or wearable device.^[16–19]^ The efficacy of mobile devices for evaluating the everyday lifestyle of a patient and the effects of digital healthcare applications on the outcomes of colorectal cancer surgery have not been reported to date.

The aim of our trial, as described herein, will therefore be to test a smartphone healthcare application in colorectal cancer patients who had received a curative surgical resection through an assessment of the HRQOL of these cases by questionnaire and by testing various metabolic parameters to evaluate whether there had been improvements to patient care.

## 2. Methods

### 2.1. Study design

A randomized controlled trial design was proposed for a planned follow-up period of 18 months post-surgery. Colorectal cancer patients of both sexes, with nationwide residences, were recruited from our university affiliated 2700-bed referral medical center. A total of 320 patients were to be enrolled and will visit their local outpatient clinics every 6 months up to 18 months. This study was approved by the Institutional Review Board of Asan Medical Center (IRB no. 2020-1015) and is reported according to the SPIRIT guidelines.^[20]^ Informed consent will be obtained from all participants by participating researchers. The patients were informed of the detailed study objectives and invited to voluntarily participate. This study is registered in the Clinical Research Information Service of Republic of Korea (KCT0005447). The study was be audited by the IRB of Asan Medical Center during the enrollment period.

### 2.2. Selection criteria

**Figure 1.**
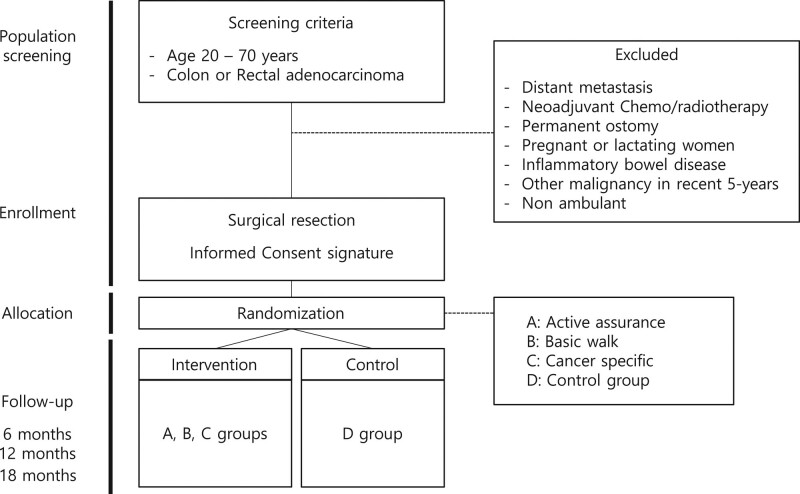
Flowchart of screening and inclusion process.

Patients diagnosed with colorectal cancer from November 2020 to November 2021 were included in the initial screen if they are aged between 20 and 70 years; had a pathologically diagnosed adenocarcinoma in the colon or rectum; and had undergone a surgical resection with curative intent. Patients were not eligible if a distant metastasis is present, if they are candidates for neoadjuvant treatment (either chemotherapy or radiotherapy), had a planned permanent ostomy, are pregnant or lactating, have been diagnosed with inflammatory bowel disease or with a malignancy in another organ within the previous 5 years, or if they are non-ambulant (Fig. 1).

### 2.3. Randomization and intervention

**Figure 2.**
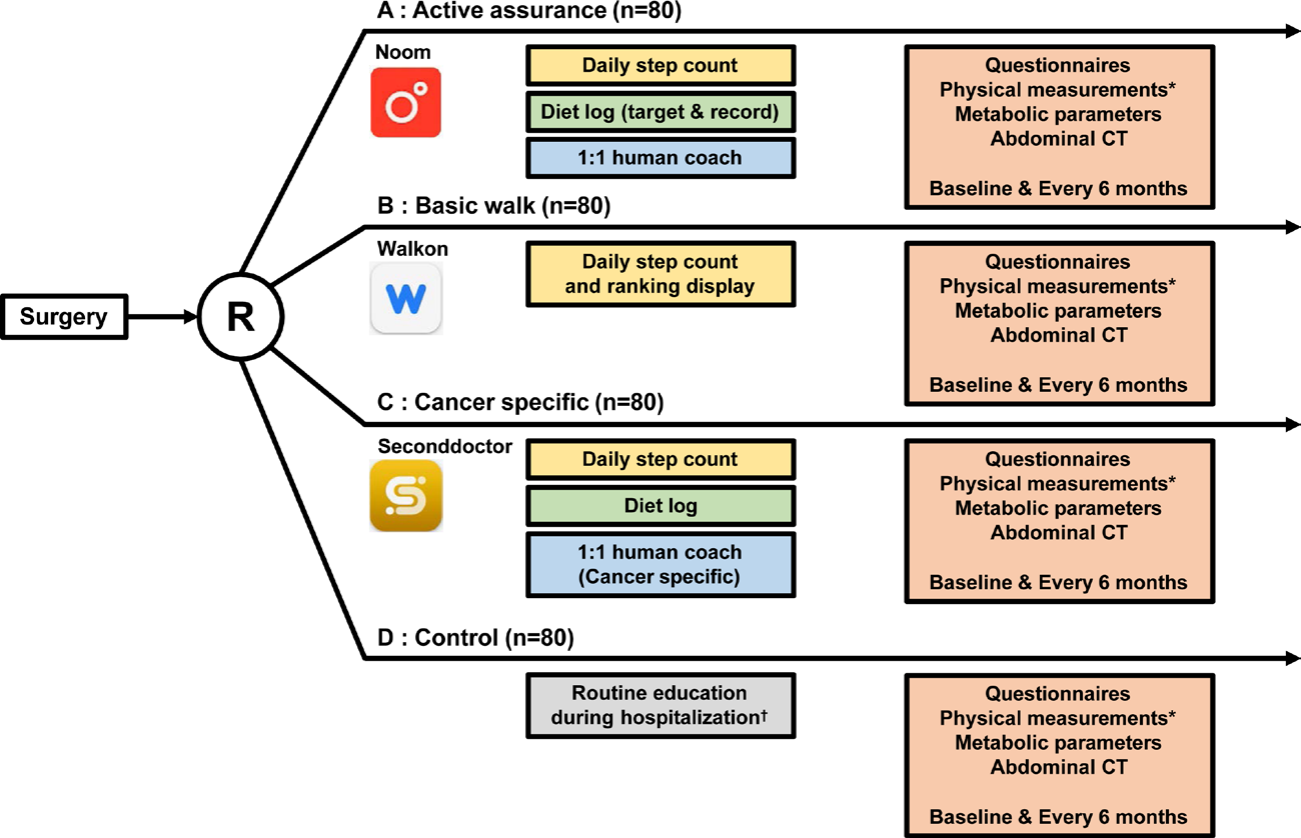
Schematic flowchart of clinical trial describing functions of each application and patient assessment during study progress. *Physical measurements include weight, height, and waist circumference; Metabolic parameters include fasting glucose, HbA1c, triglyceride, and HDL cholesterol. †Routine education during hospitalization consists of a single education of recommended diet for the post-operative 30 days. HbA1c = hemoglobin A1C, HDL = high-density lipoprotein.

After population screening were conducted for eligible patients and the appropriate cases were enrolled into the study cohort, the patients were assigned with computer-generated random numbers in equal numbers to 4 study groups. Screened patients were sequentially numbered until intervention groups was assigned. Informed consents were sought after surgery to minimize patient dropouts caused by an incidental intraoperative metastasis. Stratified randomizations were then performed according to age (≤40 years vs >40 years) and sex. Group A (active assurance), B (basic walk), C (cancer-specific) are the intervention groups, each matched with a different type of mobile health application, and Group D is the control group (Fig. 2). All participants are able to request discontinuation from study at any period. The adherence to intervention will be monitored by the login history of each application.

The active assurance group (Group A) is assigned to the Noom application, a paid global mobile application (Noom, Inc., New York, NY). Patients can use Noom to record their basic information such as age, sex, height, and weight and set their target body weight. The Noom user can continuously record their body weight, diet pattern, exercise pattern and both human advisors and artificial intelligence use this information to provide supervision to the user on a regular basis through text messages. The basic walk group (Group B) is assigned to WalkOn (Swallaby Co., Ltd., Seoul, Republic of Korea) which is a free application that can record the step counts and walking intensity of the user. Through this application, users can check their ranking among others and can form application-based communities with other users for further motivation. The cancer specific group (Group C) is assigned to Second doctor (Medi Plus Solution C., Ltd., Seoul, Republic of Korea). This is another paid application specifically aimed at cancer patients. Users select their specific condition (e.g., colorectal cancer) and upload basic information along with information related to the treatment received. One-on-one consultations are available with human coaches through this application including nutritionists and patients can automatically record their daily step count, consumed calories, heartbeat, and sleep patterns using a smart band.

### 2.4. Data collection and study outcomes

Clinicopathologic data including age, sex, morbidities, tumor location, type of operation received, pathologic stage, and adjuvant treatments will be collected from the electronic medical records of the study participants at the time of enrollment and again every 6 months until the end of the study. Surveys evaluating the HRQOL will be conducted via self-reporting paper-based questionnaires. Physical measurements, metabolic parameters (fasting glucose, hemoglobin A1C, triglyceride, high-density lipoprotein cholesterol), and fat/muscle mass measured by abdominal computed tomography (CT) will be checked prior to surgery and every 6 months after surgery for 18 months. Body composition on CT will be evaluated with artificial intelligence software (AID-U™, iAID Inc, Seoul, Korea), a fully automatic deep learning system for both third lumbar vertebra (L3) selection and body composition assessment.^[21]^ Two experienced operators (YK and KWK) will check the quality of segmentation results in all L3 level segmentation images. Skeletal muscle area including all muscles on the selected axial images, that is, psoas, paraspinal, transversus abdominis, rectus abdominis, quadratus lumborum, and internal and external obliques, the visceral fat area, and the subcutaneous fat area will be measured. All medical data will be maintained in an electronic case report form. Each application will provide data regarding patient login histories to assess compliance with the use of the mobile application. Each patient will be assigned a unique identification code to ensure the security of all personal information and data will be stored in a password-protected file that can only be accessed by authorized researchers. The timeline of data collection is presented in Table 1.

**Table 1:** Timeline of data collection.

	Screening and enrollment index date	Visit 1 6 months	Visit 2 12 months	Visit 3 18 months
Medical history	●			
Abdominal CT	●	●	●	●
Informed consent	●			
Metabolic parameters	●	●	●	●
Questionnaires	●	●	●	●

CT = computed tomography.

The primary endpoint will be the HRQOL assessed using the EuroQol 5-dimension (EQ-5D). Secondary endpoints will be HRQOL calculated from other multiple surveys including Health-related Quality of Life Instrument with 8 Items (HINT-8), 12-Item Short Form Survey (SF-12), and Functional Assessment of Cancer Therapy-Colorectal, metabolic parameters such as blood pressure, body mass index, muscle mass, fat mass, and waist circumference, and any changes to each of these outcomes during the study period.

### 2.5. Statistical analysis

Sample size was calculated based on the difference of EQ-5D scores after 6 months of study. Prior study on HRQOL of breast cancers presented an EQ-5D index of 0.86 (standard deviation [SD] 0.1) at 6 months compared to 0.95 (SD 0.08) of healthy age-matched population. Considering this, we hypothesized that significant difference of EQ-5D at 6 months to be 0.05.^[22]^ Patients are classified into 4 groups and any type 1 errors were corrected accordingly. The type 1 error margin was set at 0.008 in accordance with the study design and, considering the power of 0.8, precalculations estimated that each group would require at least 63 patients to detect significant difference. With the expected dropout rate in clinical trials of about 20%, the required sample size was calculated to be 80 patients for each group (63*100/80 = 78.75).

Intention-to-treat analysis and per-protocol analysis will be utilized to compare the intervention groups and control group. Variables and outcomes will be analyzed using either a chi square or Fischer’s exact test. Continuous data will be analyzed using a Student *t* test or Mann–Whitney *U* test, as appropriate. The changes at 6-, 12-, and 18-months post-surgery will be assessed against the baseline values and compared between the intervention and control groups using a linear mixed model. The changes in outcome values among the 3 intervention groups will be compared using repeated measures ANOVA analysis. All continuous variables will be expressed as mean values with SD, and *P* values < 0.05 will be considered to indicate statistical significance. Patients with incomplete data will still be included in the analysis. All statistical analyses will be performed using SPSS® version 21.0 (IBM, Armonk, NY).

## 3. Discussion

Colorectal cancer is considered a marker of socioeconomic development and countries transitioning to a more developed economic status have tended to show a uniform increase in its incidence.^[23,24]^ This phenomenon is also taking place in the Republic of Korea in which the incidence rate of CRC ranked second highest worldwide in 2018.^[25]^ Improving the HRQOL in CRC survivors after surgery is essential for a healthier and happier subsequent lifestyle, but is also an important consideration in terms of oncological outcomes (Fig. 2).^[9,10]^ Each patient will have different life circumstances including their basic physical status and living habits. Hence, collecting individualized information to provide specific medical advice is a necessity. To achieve this, an influx of studies has suggested the use of digital devices (internet, smartphones, watches, etc) and many report promising results from this in terms of providing supportive care for cancer patients.^[16,17,26]^

Choosing the most effective modality to collect data is important in this context. Internet-mediated interventions for patients have been reported not to be very effective as there is often a waning adherence to the use of a website over time.^[27,28]^ On the other hand, health-related applications on smart phones are repeatedly reported to be efficient in communicating between medical providers and patients.^[16–19]^ In a recent study, a mobile application was found to be an effective tool for increasing physical activity in breast cancer survivors.^[17]^ The authors of that report tested the efficacy of the “Walkon” application which we will also test in our present trial to obtain daily step counts and also application-based questionnaire responses to evaluate patient distress. The aforementioned study in breast cancer survivors found that the Walkon application significantly increased the steps by 8683.4 per week (*P* < .0001) and decreased the distress scores by 0.77 per week (*P* = .009).^[17]^ The same author group also reported excellent compliance rates of up to 88% using smartphone applications in another prior report.^[16]^ This was encouraging to us as mobile devices have also been proven to be effective in managing chronic conditions such as diabetes and heart disease, but studies of their use in managing cancer patients have remained limited.^[29]^ In addition, previous studies based on mobile interventions have typically limited their focus on meeting the needs for symptom-related information. There have been no such studies to date that have evaluated these tools for comprehensive cancer-related information, that is, from psychological support to managing cancer treatments, and the possible long-term survival benefits of using health-related mobile applications.

Through the present proposed study, we aim to assess the effects of 3 different mobile health applications in a prospective CRC cohort. In the Active assurance group, physical activity levels, dietary intake, and physical parameters (weight, body mass index) will be recorded, and the patients will be provided with one-to-one online human coaching regarding their dietary control and exercise. In the Basic walk group, daily physical activity will be recorded and activity scores will be ranked among the application community. However, no direct human consultations will be given. The Cancer specific group data will be generated in a similar manner to the Active assurance group but more specific information regarding the patients’ colorectal cancer will be collected during a one-on-one consultation with a clinical practitioner. All of the patients in our trial will be evaluated in a comprehensive manner using questionnaires to assess for any psychological distress and using abdominal CT scans to make objective measurements such as muscle mass. This study will thereby report the effectiveness of mobile applications for CRC patient management after resection surgery compared to current routine practices and provide detailed results from different health-related applications that are already in current use.

## Author contributions

Initial draft of the protocol: YIK. Revision of the protocol: YL, HK, SC, C-MC, HJL, KWK, YK, S-CY, M-WC. Final approval of the manuscript: JWL, IJP, CWK, YSY, S-BL, CSY, JCK. All authors read and approved the final manuscript.

**Conceptualization:** Yura Lee, Jong Won Lee.

**Data curation:** Yura Lee, Kyung Won Kim, Yousun Ko.

**Formal analysis:** Sung-Cheol Yun, Min-Woo Jo.

**Funding acquisition:** Jong Won Lee.

**Investigation:** Hui Jeong Lee.

**Project administration:** Hui Jeong Lee, Jong Won Lee.

**Resources:** Chan Wook Kim, Yong Sik Yoon, Seok-Byung Lim, Chang Sik Yu, Jin Cheon Kim, Kyung Won Kim, Yousun Ko.

**Supervision:** In Ja Park, Chan Wook Kim, Yong Sik Yoon, Seok-Byung Lim, Chang Sik Yu, Jin Cheon Kim, Seockhoon Chung, Sung-Cheol Yun, Min-Woo Jo, Jong Won Lee.

**Writing – original draft:** Young Il Kim.

**Writing – review & editing:** Young Il Kim, In Ja Park, Harin Kim, Chang-Min Choi.
